# Developmental regulation of *Dirofilaria immitis* microfilariae and evaluation of ecdysone signaling pathway transcript level using droplet digital PCR

**DOI:** 10.1186/s13071-020-04480-w

**Published:** 2020-12-09

**Authors:** Tsai-Chi Shang Kuan, Roger K. Prichard

**Affiliations:** Institute of Parasitology, McGill University, Sainte-Anne-de-Bellevue, Montreal, H9X3V9 Canada

**Keywords:** *Dirofilaria immitis*, Microfilaria, *Anopheles gambiae*, Ecdysone signaling pathway, 20-Hydroxyecdysone

## Abstract

**Background:**

Current measures for the prevention of dirofilariasis, caused by the dog heartworm, *Dirofilaria immitis*, rely on macrocyclic lactones, but evidence of drug-resistant isolates has called for alternative approaches to disease intervention. As microfilariae are known to be in a state of developmental arrest in their mammalian host and then undergo two molts once inside the arthropod, the aim of this study was to look at the developmental regulation of *D. immitis *microfilariae that occurs in their arthropod host using* in vitro* approaches and to investigate the role of the ecdysone signaling system in this development regulation.

**Methods:**

*Dirofilaria immitis* microfilariae extracted from dog blood were incubated under various culture conditions to identify those most suitable for* in vitro* culture and development of the microfilariae, and to determine the effects of fetal bovine serum (FBS), mosquito cells, and ecdysteroid on the development of the microfilariae. Transcript levels of the ecdysone signaling pathway components were measured with droplet digital PCR (ddPCR).

**Results:**

*In vitro* conditions that best promote early development of *D. immitis* microfilariae to the “late sausage stage” have been identified, although shedding of the cuticle was not observed. FBS had inhibitory effects on the development and motility of the microfilariae, but media conditioned with *Anopheles gambiae* cells were favorable to microfilarial growth. The transcript level study using ddPCR also showed that ecdysone signaling system components were upregulated in developing microfilariae and that 20-hydroxyecdysone increased the proportion of larvae developing to the sausage and late sausage stages* in vitro*.

**Conclusions:**

The arthropod host environment provides cues required for the rapid development of* D. immitis* microfilariae, and the ecdysone signaling system may play an important role in filarial nematode developmental transitions. This study contributes to a better understanding of the developmental process of *D. immitis* microfilariae.
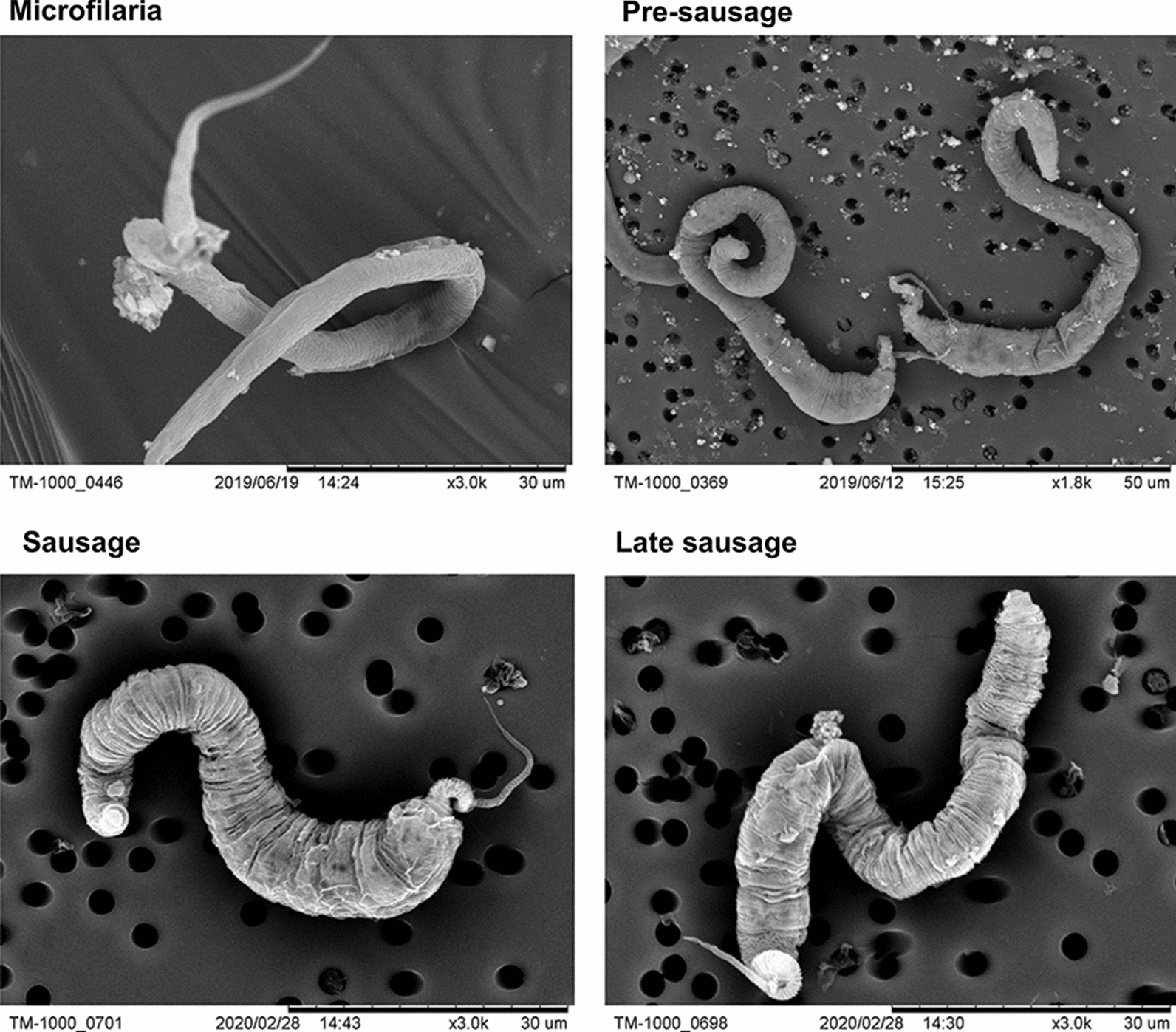

## Background

*Dirofilaria immitis,* also called the dog heartworm, is the causative agent of cardiovascular dirofilariasis, a serious and potentially fatal disease in pets that is spread by mosquitoes. All canids, regardless of age or sex, are highly susceptible to heartworm infections [1]. According to the 2019 American Heartworm Society (AHS) incidence survey, the total number of cases of dogs with heartworm disease reported in the USA has risen by about 12% since the organization’s first survey 18 years ago. Prophylaxis relies heavily on macrocyclic lactones (ML), which mainly target the third- and fourth-stage larvae, but long-term use of ML has led to the development of drug-resistant isolates, challenging current therapeutic control [2–8]. Studying the regulation of other developmental stages of the nematode thus provides an attractive approach to identifying alternative therapeutic targets.

*Dirofilaria immitis* requires an arthropod and a mammalian host to complete its life cycle. Inside the mammalian host, adult female worms residing in the pulmonary vasculature can release high numbers of microfilariae into the circulation after mating with the male worms [9]. These microfilariae remain in a state of developmental arrest in the bloodstream for up to 2.5 years until an arthropod vector ingests them [10, 11]. A wide range of mosquito species can be exploited by the parasite [12, 13]*.* Upon being taken up by a mosquito, microfilariae will migrate from the midgut to the Malpighian tubules, where they quickly undergo developmental transitions known as molts, or the shedding of the old cuticle. At temperatures between 23 °C and 27 °C, the first molt, in the mosquito, usually takes place between 8 and 10 days post-infection and causes the stoma to open to the exterior. The second molt to the third-stage (L3) larvae takes place around 2–3 days after the first molt [14–17]; this molt removes the anal plug [18, 19]. Our current knowledge of these stages indicates that the developmental cues that trigger molting are closely tied to the transition to a new host environment. Inhibition of the molting process would thus arrest the life cycle and prevent the pathology of dirofilariasis.

Similar to nematodes, arthropods are ecdysozoans that develop through a series of periodic larval stage molts before reaching adulthood [20]. Key pathways that regulate the growth of insects have been well-characterized: cell and tissue growth rates are known to be controlled by the insulin/insulin-like growth factor signaling pathway, whereas developmental transitions and metamorphosis are under the control of the ecdysone signaling pathway [21]. Ecdysteroids are sterol derivatives that bear similar structural features and are synthesized in arthropods from dietary cholesterol upon metabolism by cytochrome P450 enzymes. 20-Hydroxyecdysone (20E), the active ecdysteroid hormone that regulates the physiological and behavioral changes during insect molting [22], is converted from ecdysone by the ecdysone 20-monooxygenase in peripheral tissues, such as the fat body, ovaries, midgut, and Malpighian tubules [23–26]. The hormonal action of 20E is exerted through binding to the ecdysone receptor (EcR), a heterodimeric nuclear receptor (NR) composed of EcR and the ultraspiracle protein (USP), the insect orthologue of the mammalian retinoid X receptor protein (RXR) [27–29]. Upon activation, EcR/USP directly activate a cascade of gene expression by recognizing the ecdysone-response element (EcRE) 5′–(A/G) G (G/T) T C A N T G A (C/A) C (C/T)–3′ in the promoter region of its target genes [30, 31]. According to the Ashburner model [32, 33], this leads to the expression of a set of early regulatory genes (at least six in *Drosophila melanogaster*, including *E74*, *E75*, *E78*, *HR3*, *HR4*, and *βFtz-F1*) [34], but represses the expression of the late regulatory genes. The proteins of the early genes then repress their own promoters while inducing the expression of the late genes, whose protein products play a more direct role in controlling molting and metamorphosis.

As in insects, a possible candidate pathway that controls molting in filarial nematodes is the ecdysone signaling pathway. Both free and conjugated ecdysteroids have been detected in the larval and adult stages of several species of filarial nematodes, including *D. immitis* and *Onchocerca volvulus* [35, 36]. Elements of the ecdysone signaling pathway and the NR that function downstream of EcR and USP have also been identified in *D. immitis* [37–40]. In addition, evidence for a functional ecdysone signaling system in filarial nematodes was also demonstrated in *Brugia malayi* in a study by Tzertzinis et al. [41]. When exogenously applied, ecdysteroids have been shown to exert biological effects on the molting of third-stage and fourth-stage nematodes. For example, low concentrations of 20E were found to stimulate the molting of *D. immitis* [42] and *Ascaris suum* third-stage larvae [43], as well as *Heligmosomoides polygyrus* fourth-stage larvae [44]. In addition, non-steroidal ecdysteroid agonists (RH compounds) were also found to induce the molting of third-stage larvae of *D. immitis* [42]. Although the role that the ecdysone signaling pathway plays in the developmental transition of third-stage and fourth-stage larvae is evident, the role of ecdysteroids in microfilarial development is largely unknown.

This study attempts to establish an* in vitro* culture condition similar to the mosquito host environment to determine factors that stimulate the development of *D. immitis* microfilariae and to identify the role of the ecdysone signaling system during the developmental process. This work advances understanding of *D. immitis* microfilaria* in vitro* development and offers insights to novel targets for drug development.

## Methods

### *In vitro* culture of *D. immitis* microfilariae

Microfilariae of *D. immitis* (2005 Missouri strain) were provided by FR3 Molecular Resources through the BEI Resources Repository [45]. Blood samples containing microfilariae were collected from infected dogs and shipped overnight to McGill University. Upon arrival, the blood samples were diluted with NaHCO_3_ (Sigma-Aldrich, St. Louis, MO, USA) and filtered through 3.0-µm polycarbonate membrane filters as previously described [46]. After several washes in phosphate buffered saline (PBS), microfilariae were plated in a 24-well plate at a density of 500 microfilariae per well in culture media supplemented with 100 U/ml penicillin (Gibco™; Thermo Fisher Scientific, Waltham, MA, USA ), 100 µg/ml streptomycin (Gibco™), 0.25 µg/ml amphotericin B (Gibco™), and 0.01 mg/ml gentamicin (Sigma-Aldrich). The media tested included RPMI-1640 with l-glutamine (Gibco™), Dulbecco’s modified Eagle’s medium (DMEM) with high glucose and l-glutamine (Gibco™) levels, Ham’s F-12 (Nutrient Mix) with l-glutamine (Gibco™), DMEM and Ham’s F-12 mixed at a 1:1 ratio, and Schneider’s insect medium with l-glutamine (Sigma-Aldrich). The parasites were incubated at 26 °C in 5% CO_2_ for 22 days, with/without the presence of *Anopheles gambiae* cells or fetal bovine serum (FBS). To test the effects of serum, 10% (v/v) heat-inactivated FBS (Gibco™) was added to each well.

To assess the effects of ecdysteroid on the development of microfilariae* in vitro*, microfilariae were incubated in DMEM and Ham’s F-12 mixed at a 1:1 ratio without *An. gambiae* cells or conditioning with these cells. 20E (Cayman Chemical Co., Ann Arbor, MI, USA) dissolved in DMSO was added to the incubation at a final concentration of 5 µM, 0.05% (v/v) DMSO. To increase the possible uptake of 20E to the *D. immitis* larvae, 0.004% (v/v) (2-hydroxypropyl)-β-cyclodextrin (Sigma-Aldrich) was included in the control and 20E incubations.

### *Anopheles gambiae* cell culture

*Anopheles gambiae* cell line 4a-3B (provided by Dr. George K. Christophides) was maintained at 26 °C in 5% CO_2_ in Schneider’s insect medium supplemented with 10% (v/v) heat-inactivated FBS, 100 U/ml penicillin, and 100 µg/ml streptomycin. For co-culture with *D. immitis* microfilariae, *An. gambiae* cells were seeded at a density of 5 × 10^4^ cells/cm^2^ 48 h prior to co-culturing and washed with PBS 24 h later. The cells were then incubated in the culture medium intended for culturing the parasite for the remaining 24 h prior to introducing the microfilariae. Cells were re-seeded every 3 days. To condition media with *An. gambiae* cells, cells were seeded at the same density as mentioned above, and after 24 h they were washed with PBS and incubated in the culture medium for another 24 h. Only the cell culture medium was collected and used for *D. immitis* culture.

### Development of *D. immitis* microfilariae

Microfilariae were observed every 24 h during the 22-day incubation, and culture media were renewed every day with the respective fresh complete medium. Body dimensions of the parasites were measured and analyzed with the Nikon NIS-Elements Advanced Research image analysis software (Nikon Corp., Tokyo, Japan). Morphological changes in the parasites were noted, and the motility of the parasite was measured as any movement within a 30-s timeframe. The percentage of each stage of development was calculated as: (mean of stage-specific parasite from a triplicate or quadruplicate assay/total number of microfilariae) × 100.

The IncuCyte live-cell imaging and analysis platform was used for the live worm analysis. Live microfilariae were cultured in 24-well cell culture plates at 26 °C, 5% CO_2_ and imaged with the IncuCyte ZOOM system (Essen BioScience, Royston, Hertfordshire, UK) at 20× magnification.

### Scanning electron microscopy

Microfilariae were pre-chilled at 4 °C for 30 min and placed in fixative of 2.5% glutaraldehyde and 1.5% formaldehyde in PBS for 2 h. The samples were then washed with PBS (3 × 10 min) and dehydrated in an ethanol series of 30% (1 × 10 min), 50% (1 × 10 min), 70% (1 × 10 min), 80% (1 × 10 min), 90% (1 × 10 min), and 100% (3 × 30 min). Dehydrated samples were dried in a critical point dryer by CO_2_ treatment (Leica EM CPD300; Leica Microsystems, Wetzlar, Germany) or chemically dried with hexamethyldisilazane (HMDS) (Sigma-Aldrich) (3 × 15 min). Dried samples were sputter-coated with 4 nm of gold and palladium using the Leica EM ACE200 coater (Leica Microsystems) and mounted onto aluminum stubs for scanning electron microscopy (SEM) observation (Hitachi TM-1000 EM microscope; Hitachi Ltd., Tokyo, Japan).

### Quantification of transcript level

Adult *D. immitis* samples (Kentucky strain, ML-susceptible strain) were provided by Zoetis Inc. (Parsippany-Troy Hills, NJ, USA) for the transcript-level study. The adult worms were obtained through necropsy of experimentally-infected dogs and flash-frozen upon collection.

Total RNA from 6–8 × 10^4^ *D. immitis* microfilariae (Missouri strain) and individual male or female adult worms (Kentucky strain) was isolated with TRIzol™ reagent (Ambion, Invitrogen, Thermo Fisher Scientific), following the manufacturer’s protocol. Homogenization was performed by crushing the parasite using plastic pestles and 425- to 600-µm glass beads (Sigma-Aldrich). Extracted RNA was treated with DNase using an Invitrogen DNA-free kit (Thermo Fisher Scientific). RNA concentration was assessed with a NanoDrop One^C^ spectrophotometer (Thermo Fisher Scientific), and the quality determined by running an aliquot of the RNA sample on a native agarose gel. cDNA was obtained by reverse transcription using the SuperScript III first-strand synthesis system (Thermo Fisher Scientific) with oligo (dT)_20_ primers.

Detection of target genes and validation of primers (Additional file 1: Table S1) were performed with PCR and quantitative (q) PCR using *Taq* DNA polymerase (Invitrogen, Thermo Fisher Scientific) and 2× SYBR Select Master Mix (Applied Biosystems, Foster City, CA, USA), respectively. The absolute quantification of target nucleic acids present in the sample was determined by droplet digital PCR (ddPCR). In brief, the ddPCR mix comprised 2× QX200 ddPCR EvaGreen Supermix (Bio-Rad, Hercules, CA, USA), 250 nM final concentration of each forward and reverse primer, cDNA sample, and nuclease-free water. Droplet generation oil (Bio-Rad) was added to the ddPCR mix and the mixture then partitioned into 20,000 nanoliter-sized droplets by a QX200™ Droplet Generator (Bio-Rad). The droplets were then subjected to standard PCR amplification and read with a QX200™ Droplet Reader (Bio-Rad) to determine the target concentration using Poisson’s statistics [47]. Results were analyzed by normalizing the copy number of the gene of interest against the geometric mean of the copy numbers of three reference genes. The fold change in transcript level was then calculated by comparing the normalized value to the baseline (day 1) transcript level.

### Statistical analysis

All experiments were performed with at least three individual replicates. Data presented for the* in vitro* culture of *D. immitis* are shown as mean data ± standard deviation (SD). The effects of *Anopheles gambiae* cells on the proportion of the microfilariae developing to a later stage* in vitro* and on the proportion of each stage that were seen to be motile were analyzed by Kruskal–Wallis H-test with Dunn’s multiple comparisons test. The effects of 20E on the proportion of the microfilariae developing to a later stage* in vitro* and the proportion of each stage that were seen to be motile were analyzed by Mann-Whitney U-test, two-tailed *P *value, and 95% confidence interval. The transcript levels of the ecdysone signaling system genes were analyzed by unpaired t-test with Welch’s correction, two-tailed *P *value, and 95% confidence interval. All analyses was performed using Prism 6.0c (Graph Pad Software, Inc., San Diego, CA, USA).

## Results

### Microfilariae develop into pre-sausage, sausage, and late sausage stages

**Fig 1.**
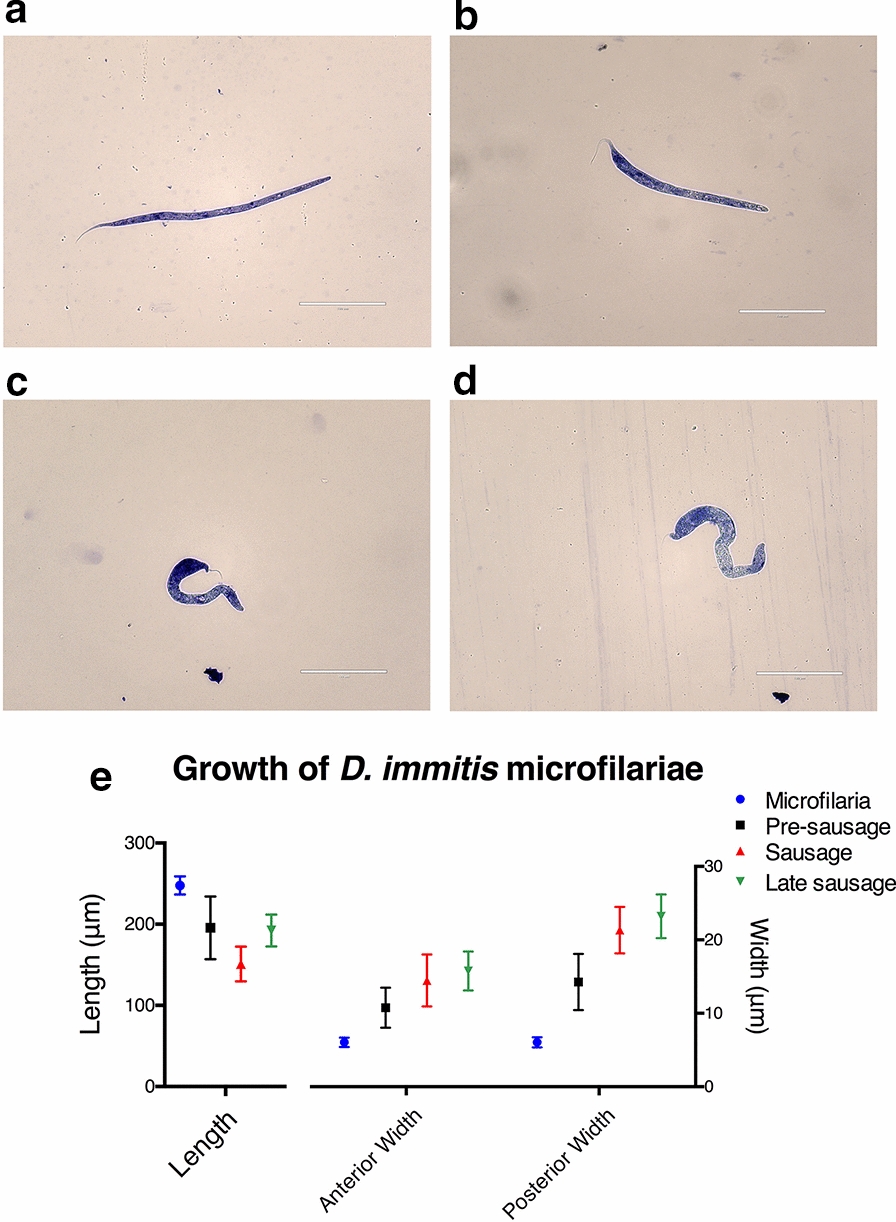
Morphological development and body dimensions of *Dirofilaria immitis* (dog heartworm) microfilariae. **a**–**d** Light microscopy images of methylene blue-stained microfilaria (**a**), pre-sausage (**b**), sausage (**c**), late sausage (**d**) parasites, at 40× magnification. **e** Mean body dimensions of microfilariae developmental stages. Data are reported as the mean with standard deviation (SD) from ten counts

As *D. immitis* microfilariae develop, they progress into morphologically distinct forms that can be categorized as microfilaria, pre-sausage, sausage, or late sausage stage (Fig. 1a–d). When first extracted from dog blood samples, parasites were in the microfilaria stage and appeared elongated and slender, with vigorous serpentine movements (Additional file 2: Video S1). When microfilariae were incubated* in vitro*, the body of the parasite shortened and the posterior region began to enlarge after several days, making the tail more distinct; this stage was classified as the pre-sausage stage. Compared to the microfilaria stage, the pre-sausage stage was more granular in appearance, and parasite movement was slowed (Additional file 3: Video S2). Over the next few days, the pre-sausage stage larvae grew shorter in length, resulting in a stumpy appearance with a fine tail at the tip, approaching the sausage stage. The sausage stage was more basophilic than the microfilaria, with heavy internal granulation, and movement was sluggish (Additional file 4: Video S3). At the late sausage stage, movement of the parasite continued to decline (Additional file 5: Video S4), and at the same time the anterior region of the sausage stage larvae started to enlarge while the body became more elongated. The length and width of the parasites were measured to determine the mean body dimensions for the different stages (Fig. 1e).

### Pre-sausage, sausage, and late sausage stage parasites are first-stage larvae

**Fig 2.**
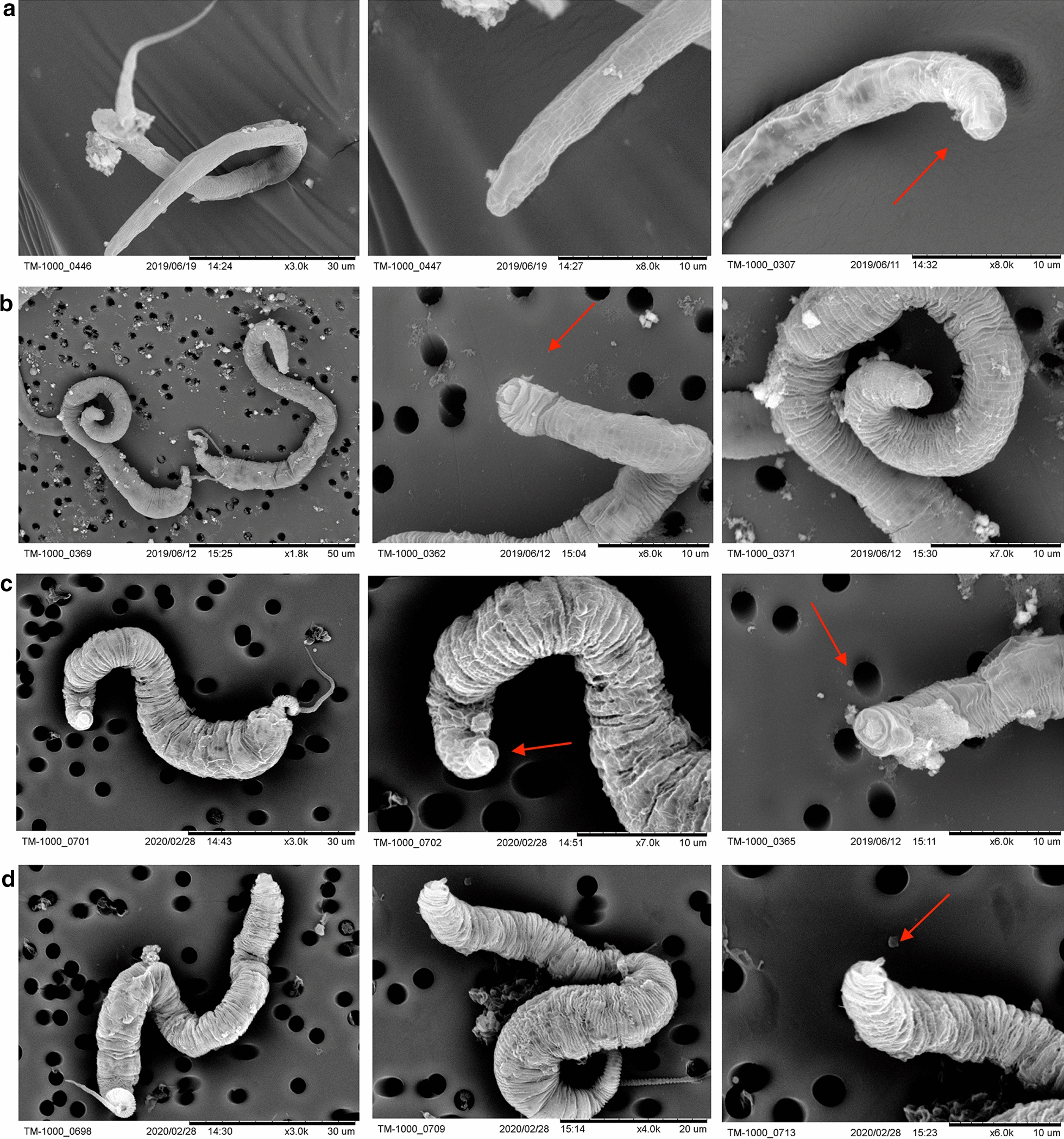
Scanning electron miscropy analysis of *D. immitis* microfilaria, pre-sausage, sausage, and late sausage stages. **a** Microfilaria mounted on a double-sided tape showing whole parasite (left), enlarged head region (middle), ventral anterior view (right). **b** Pre-sausage stage larvae mounted on a 3.0-µm polycarbonate membrane filter showing whole parasites (left), enlarged head region (middle and right). **c** Sausage stage larvae mounted on a 3.0-µm polycarbonate membrane filter showing whole parasite (left) and enlarged head region (middle and right). **d** Late sausage stage larvae mounted on a 3.0-µm polycarbonate membrane filter, showing whole parasite (left and middle) and enlarged region (right). Red arrows show where the stoma opening would be

To further study the morphological differences between the microfilaria, pre-sausage, sausage, and late sausage stage parasites and to observe possible signs of molting, SEM analysis and live-cell analysis were performed. Numerous transverse grooves in the cuticle were evident in all developmental stages, giving the parasite a striated appearance (Fig. 2a–d). The grooves seemed to become more noticeable as the parasite became more developed. No visible difference was seen in the stoma region among the four stages; all of the parasites had a circular tissue located at the tip of the head and layers of folded tissue that surrounded it. Results of the live-cell analysis with the IncuCyte ZOOM system also showed that development to the late sausage stage was not accompanied by any shedding of the cuticle (Additional file 6: Video S5), although separation of the outer cuticle could be seen.

### DMEM/Ham’s F-12 medium provides a relatively favorable condition for the* in vitro* cultivation of *D. immitis* microfilariae

**Fig 3.**
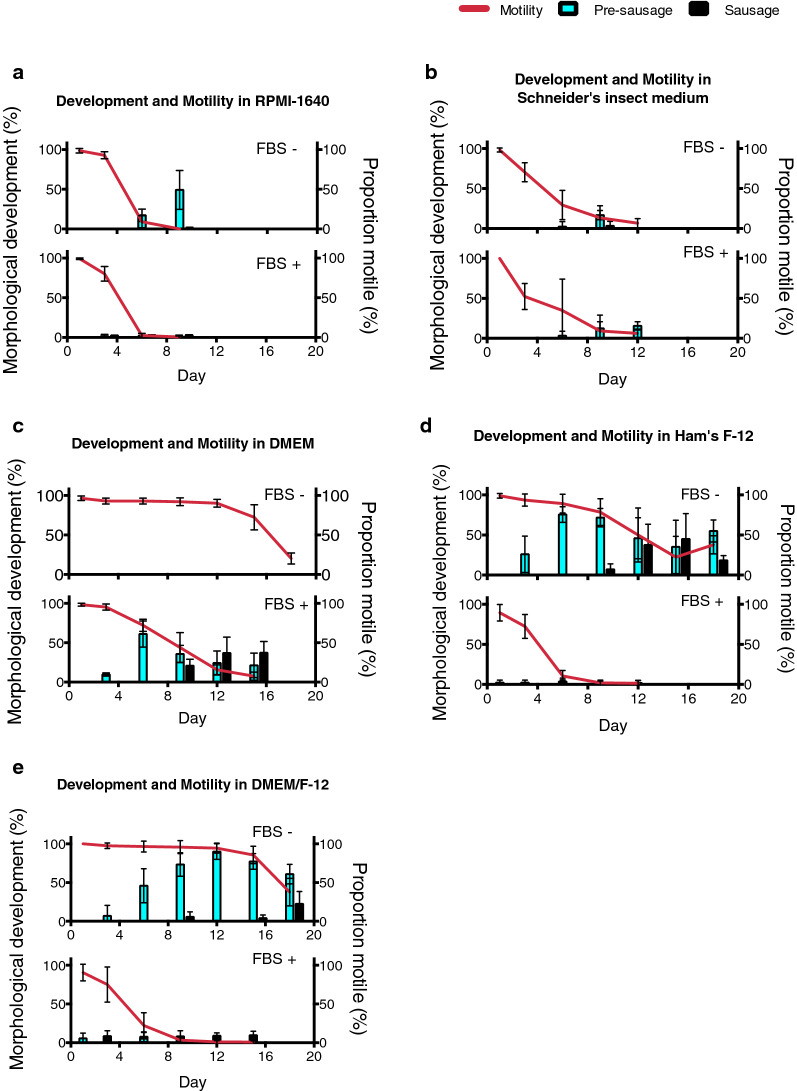
Development of *D. immitis* microfilariae and proportion of motile larvae in commercially available media and fetal bovine serum (FBS). **a** RPMI-1640 medium, **b** Schneider’s insect medium, **c** DMEM, **d** Ham’s F-12, **e** DMEM/Ham’s F-12. All data are reported as mean percentages, with error bars corresponding to the SD, from at least four individual replicates

To determine suitable conditions for the* in vitro* cultivation of *D. immitis* microfilariae, extracted microfilariae were incubated in various commercially available culture media at 26 °C for 22 days. Results show that both RPMI 1640 and Schneider’s insect medium were poor at sustaining the development and motility of the microfilariae (Fig. 3a, b). DMEM medium maintained microfilariae motility but was unable to initiate development to the pre-sausage stage (Fig. 3c), while Ham’s F-12 was beneficial to their development. Reduced motility was associated with this development (Fig. 3d). When mixed at a 1:1 ratio, DMEM/Ham’s F-12 provided a favorable condition for the microfilariae and supported larvae development to the pre-sausage and sausage stage (Fig. 3e).

When 10% heat-inactivated FBS was added to the microfilaria culture, both development and motility of the parasite were drastically reduced, and microfilariae appeared to be more lethargic and degenerate, with little development to the pre-sausage stage observed in most groups (Fig. 3a, b, d, e). An exception was seen in those that were incubated in DMEM and 10% FBS (Fig. 3c); although development was initiated in this group and some pre-sausages developed into sausages, most of the parasites appeared to be degenerated and motility was greatly reduced compared to the group without 10% FBS.

### *Anopheles gambiae* cells and cell-secreted factors promote *D. immitis* microfilariae development

**Fig 4.**
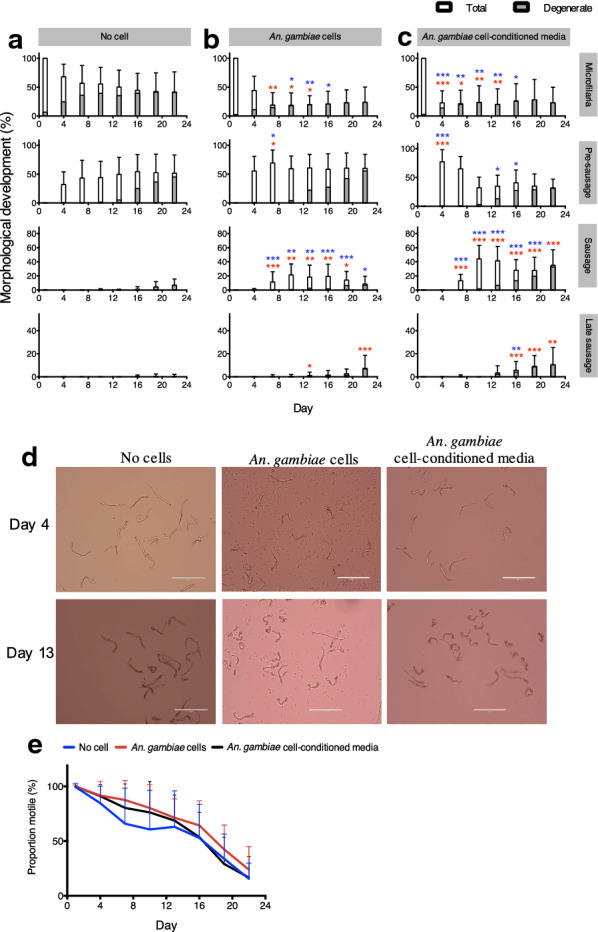
Development of *D. immitis* microfilariae cultured with *Anopheles gambiae* cells or cell-conditioned media. Results are presented as the percentages of larvae at the microfilaria, pre-sausage, sausage, and late sausage stages cultured in the absence of *An. gambiae* cells (**a**), with *An. gambiae* cells (**b**), with *An. gambiae* cell-conditioned media (**c**). All data are reported as mean percentages, with error bars corresponding to the SD from at least three individual replicates that were performed three times. The significance of the effects of *An. gambiae* cells on *D. immitis* development was analyzed by the Kruskal-Wallis H-test with Dunn’s multiple comparisons test using Prism 6.0c (Graph Pad Software, Inc.). Asterisks denote the statistical difference between total (red asterisks) and healthy (blue asterisks) parasites versus the no-cell group. *p < 0.05, **p < 0.01, ***p < 0.001. **d** Light microscopy of *D. immitis* microfilariae cultured with no cells, *An. gambiae* cells, or *An. gambiae* cell-conditioned media at day 4 and day 13. **e** Proportion of motile *D. immitis* larvae cultured in the absence of cells, with *An. gambiae* cells, or with *An. gambiae* cell-conditioned media. The significance of the effects of *An. gambiae* cells or cell-conditioned media on *D. immitis* motility was analyzed by the Kruskal–Wallis H-test with Dunn’s multiple comparisons test using Prism 6.0c (Graph Pad Software, Inc.)

*Anopheles gambiae* cells were introduced into the culture of microfilariae incubated in DMEM/Ham’s F-12 for 22 days to test the effect of insect cells on the development of the microfilariae. Consistent with results shown in Fig. 3, larvae incubated in DMEM/F-12 in the absence of mosquito cells developed into the pre-sausage stage at around day 4, but few developed further into the sausage stage throughout the 22-day incubation period. The presence of *An. gambiae* cells, however, initiated faster development, and significantly higher levels of sausage stages were observed as early as day 7 (Fig. 4a, b); also, some parasites were able to develop into the late sausage stage.

When medium conditioned with *An. gambiae* cells was used instead of having physical cells in the culture, a similar effect in the development of microfilariae was also seen. In fact, there was a significant decrease in the percentage of microfilaria and an increase in the number of pre-sausage stage larvae during early incubation. In addition, more sausage stage larvae were observed when the cell-conditioned medium was used compared to when microfilariae were in physical contact with the insect cells (Fig. 4c, d).

While motility always decreased as the microfilariae developed to the pre-sausage and later stages, the presence of cells and cell-conditioned media seemed to better maintain parasite activity, whereas in the absence of cells, motility dropped at a faster rate and the parasites appeared more sluggish. The presence of cells in the culture also appeared to slow the drop in motility compared to parasites cultured in cell-conditioned media, although the motility results were not significantly different (Fig. 4e).

### *EcR*, *rxr-1* and downstream early regulatory genes are upregulated in developing microfilariae

**Fig 5.**
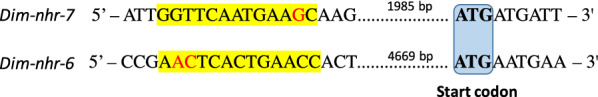
Putative ecdysone-response element (EcRE) in the promoter region upstream of the start codon of *Dim-nhr-7* and *Dim-nhr-6*, downstream early regulatory genes of the ecdysone receptor (EcR). Upstream promoter sequence of *Dim-nhr-7* nDi.2.2.2.g02617 and *Dim-nhr-6* nDi.2.2.2.g04428 [49] were screened for EcRE. Start codons are marked with the blue box, putative EcRE are highlighted in yellow, and nucleotides that differ from the canonical EcRE are highlighted in red.

**Fig 6.**
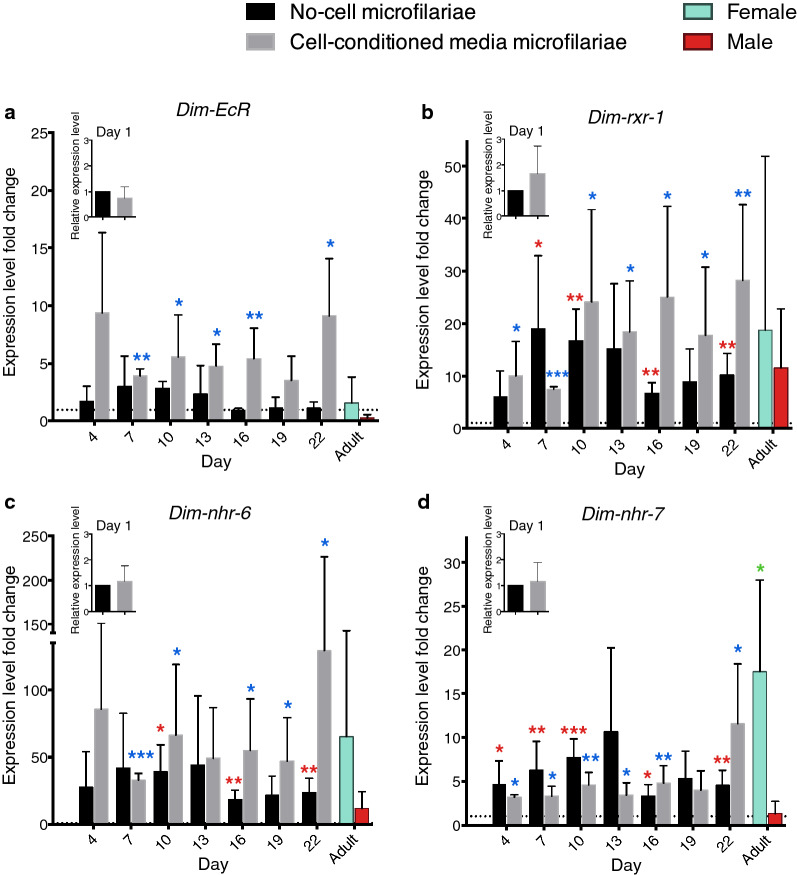
Transcript level of the ecdysone signaling system in *D. immitis* using droplet digital PCR. Fold changes for both no-cell and cell-conditioned media groups were calculated relative to day 1 of each medium. Fold changes for adults were calculated relative to day 1 of the cell-conditioned media group. Insert figures show transcript level at day 1 for both media. All transcript levels were normalized to three reference genes: *Dim-GAPDH*, *Dim-Actin*, and *Dim-β-tubulin*. **a** *Dim-EcR*, **b** *Dim-rxr-1*, **c** *Dim-nhr-6*, **d** *Dim-nhr-7*. All data are reported as mean fold change, with error bars corresponding to the SD from at least two individual replicates that were performed three times. The significance of the transcript level fold changes compared to day 1 baseline level was analyzed by the unpaired t-test with Welch’s correction, two-tailed *p *value, and 95% confidence interval using Prism 6.0c (Graph Pad Software, Inc.). Asterisks denote the statistical difference between no-cell microfilariae (red asterisks), cell-conditioned media microfilariae (blue asterisks), adults (green asterisks)* vs* day 1 groups (---). *p < 0.05, **p < 0.01, ***p < 0.001

As steroid-nuclear hormone receptor signaling plays an important role in ecdysozoan development [48], microfilariae were collected every 3 days, and the transcript levels of the *Dim-EcR*, *Dim-rxr-1*, and downstream early regulatory genes were measured to determine the transcriptional change in the ecdysteroid signaling pathway during microfilarial developmental changes. *Dim-nhr-7* and *Dim-nhr-6*, orthologues of *E78* and *E75*, respectively, are both downstream early regulatory genes of the ecdysone receptor. The putative EcRE was found in the upstream promoter regions of both *Dim-nhr-7* and *Dim-nhr-6*, indicating that *Dim-nhr-7* and *Dim-nhr-6* can potentially be regulated by EcR directly (Fig. 5). All primer pairs (Additional file 1: Table S1) were optimized for specificity and efficiency, and the quantification cycle (Cq) values from qPCR were used to estimate template concentrations for use on the ddPCR. After normalizing the genes of interest to the three reference genes *Dim-GAPDH*, *Dim-Actin*, and *Dim-β-tubulin*, *Dim-EcR*, *Dim-rxr-1*, *Dim-nhr-7*, and *Dim-nhr-6* were all upregulated to different magnitudes in developing first-stage (L1) larvae, as shown in Fig. 6a–d. In both no-cell and *An. gambiae* cell-conditioned media groups, transcript levels of all four genes consistently increased during the first few days of development. However, in no-cell groups, the transcript levels of all four genes incurred a drop at around day 13–16, following which an increase was seen again on day 19. In groups cultured in the cell-conditioned medium, a gradual increase throughout the 22-day incubation period was seen for *Dim-EcR*, *Dim-rxr-1*, and *Dim-nhr-6*, with a sharp rise on day 22. In contrast, the transcript level of *Dim-nhr-7* in the cell-conditioned medium group remained relatively constant during the early incubation period, but a sharp rise could be seen on day 22.

Overall, developing larvae in cell-conditioned medium groups expressed higher transcript levels than those that were not incubated in cell medium, with the exception being the transcript level of *Dim-nhr-7*, for which the no-cell group has a higher transcription level, but the transcript level of the cell-conditioned medium group quickly rose higher on day 22. In addition, the overall transcript level of *Dim-nhr-6* in developing microfilariae was significantly higher than those of the other three genes. Transcript levels of female and male adults were also included as a comparison, and the results showed that the transcript level of the ecdysteroid signaling system components was higher in females than in males for all four genes.

### 20-Hydroxyecdysone promotes development to sausage and late sausage stage larvae

**Fig 7.**
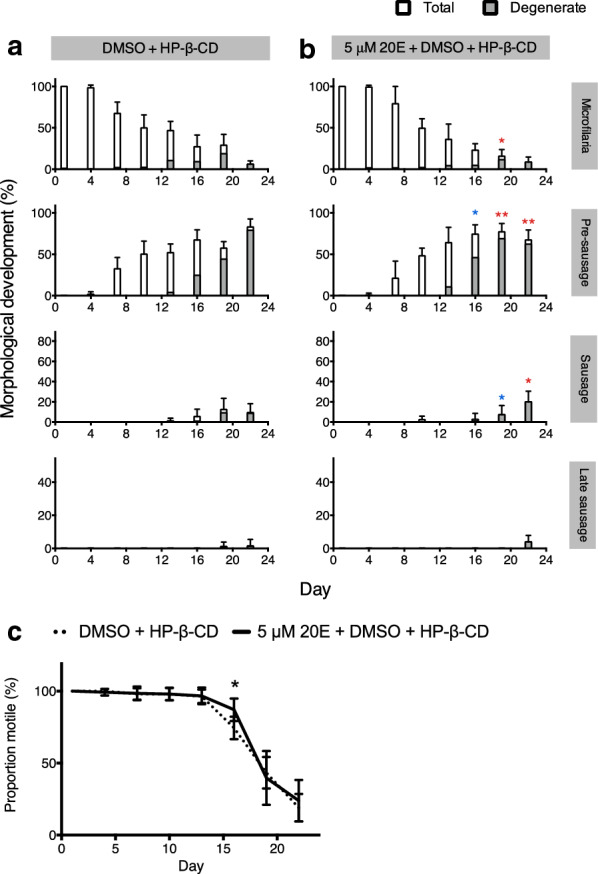
Development of *D. immitis* microfilariae treated with 20-hydroxyecdysone (HP-β-CD). Results show percentages of larvae at the microfilaria, pre-sausage, sausage, and late sausage stages cultured in 0.05% DMSO + 0.004% HP-β-CD (**a**), 5 µM 20E + 0.05% DMSO + 0.004% HP-β-CD (**b**). All data are reported as mean percentages, with error bars corresponding to the SD from at least three individual replicates. The significance of the effects of 20E on *D. immitis* development was analyzed by the Mann-Whitney U-test, two-tailed *P *value, and 95% confidence interval using Prism 6.0c (Graph Pad Software, Inc.). Asterisks denote the statistical difference between total (red asterisks) and healthy (blue asterisks) parasites* vs* DMSO + HP-β-CD group. *p < 0.05, **p < 0.01, ***p < 0.001. **c** Proportion of motile *D. immitis* larvae cultured in 0.05% DMSO + 0.004% HP-β-CD or 5 µM 20E + 0.05% DMSO + 0.004% HP-β-CD. The significance of the effects of 20E on *D. immitis* the proportion of larvae that were motile was analyzed by the Mann-Whitney U-test, two-tailed *P *value, and 95% confidence interval using Prism 6.0c (Graph Pad Software, Inc.). Asterisks denote the statistical difference between 5 µM 20E + DMSO + HP-β-CD* vs* DMSO + HP-β-CD group. *p < 0.05, **p < 0.01, ***p < 0.001

In view of the upregulation of genes involved in the ecdysone regulatory development pathway, the effects of 20E were investigated on microfilariae cultured* in vitro*, without insect cells or in insect cell-conditioned medium. When microfilariae were treated with 5 µM 20E every day, with (2-hydroxypropyl)-β-cyclodextrin (HP-β-CD) as the carrier of the hydrophobic compound, there was a significantly higher level of total pre-sausage stage larvae at day 19 and a significantly higher level of sausage stage larvae in the culture at the end of the 22-day incubation, compared to the DMSO + HP-β-CD control group. With the increase in development to the sausage stage larvae in the 20E-treated parasites compared to the control group at day 22, as might be expected there was a drop in the level of the pre-sausage stage larvae (Fig. 7a, b) at this time. There were no significant differences between the motility of parasites in the 20E-treated group and those in the control group, except that a slightly higher proportion were observed to be motile in the 20E-treated group on day 16 of incubation (Fig. 7c).

## Discussion

As there is very little publisher information on the* in vitro* development of *D. immitis* microfilariae [50–52], the aim of this research was to understand the developmental regulation underlying the development of *D. immitis* microfilariae in the hope of finding an alternative strategy to truncate the life cycle of the parasite.

### The composition of DMEM and Ham’s F-12 culture media promote the* in vitro* development of *D. immitis* microfilariae

In this study, *D. immitis* microfilariae developed into late L1 parasites that could be classified as pre-sausage, sausage, and late sausage stages. These late L1 stages differed considerably in their morphological appearances (Figs. 1, 2). However, similar to previous attempts to study the vector phase development of filarial nematodes [51–59], none of the *D. immitis* microfilariae in this study developed past the L1 stage, as no visible opening of the stoma was observed (Fig. 2) and no shedding of the cuticle took place between the transitions (Additional file 6: Video S5).

Among the commercially available culture media tested, DMEM and Ham’s F-12 medium mixed at a 1:1 ratio provided a relatively favorable condition for the* in vitro* cultivation of microfilariae. In contrast, Schneider’s insect medium and RPMI 1640 medium were both poor at sustaining parasite development and motility (Fig. 3). These findings are consistent with previous studies done by Sneller and Weinstein [50]. As motility changes with development, with microfilariae being more mobile than the more developed stages, the level of motility should not be taken as a measure of vitality. In our study, motility has been used as an indication of viability only, with motile larvae being assessed as alive and larvae that are immotile over a period of time being considered to be inactive and possibly dead. A closer look at the media composition showed that Schneider’s insect medium contains high levels of intermediate compounds of the tricarboxylic acid (TCA) cycle while lacking in most B vitamins. As some filarial nematodes have low TCA cycle enzyme activity [60–62], it is possible that intermediates of the TCA cycle do not contribute significantly to the overall energy metabolism of the parasite, while the lack of B vitamins may be detrimental to the growth and development of the microfilariae [63]. In addition, compared to DMEM and Ham’s F-12, RPMI 1640 medium is comparatively lower in terms of content of a variety of inorganic salts and lacks sources of certain ions, such as copper, iron, and zinc [64, 65], which could have contributed to the poor growth and motility of the parasite. In comparison, the high levels of glucose, vitamins [63], and amino acids [66] in DMEM may be important energy sources for sustaining parasite motility, while the diversified components in Ham’s F-12, including diamines [67], polyunsaturated fatty acid [68], organosulfur compound, purine, and pyrimidine [69], may contribute to the initiation of microfilaria development.

### Microfilaria development is driven by cues from the arthropod host cells but inhibited by mammalian-derived metabolites

The importance of mosquito cells in microfilarial cultures has been reported [52, 57, 70], but the secretion of microfilarial growth-promoting factors by mosquito cells has, to our knowledge, been demonstrated for the first time in this study. The favorable effect of mosquito cells was shown when *D. immitis* microfilariae were cultured with *An. gambiae* 4a-3B cells, where microfilariae that were cultured with mosquito cells showed early appearance of the sausage and late sausage stage parasites (Fig. 4). Because there was no observable interaction between the parasite and the cell layer underneath, we hypothesized that the growth-promoting factors of the cells may be secreted into the media. On further testing, media conditioned with *An. gambiae* cells also had a similar effect, indicating that factors secreted by the mosquito cells provided conditions similar to the environment in the mosquito and promoted faster microfilarial development.

FBS was found to inhibit the development of the microfilariae* in vitro* (Fig. 3). A possible inhibitory component of the serum could be mammalian cholesterol derivatives, such as cholestenoic acid. Cholesterol derivatives have been found to contribute to the molting of *D. immitis* L3 larvae, a stage that develops inside the mammalian host [71]. This result indicates that the presence of mammalian cholesterol metabolites may imitate the mammalian host environment, in which microfilariae would be in a state of developmental arrest. However, it remains unclear why the motility of the parasites in the presence of FBS was reduced, nor is it clear whether the resultant change in motility reflects a change in viability or not, given observations,* in vivo *and* in vitro*, that motility changes naturally with development.

### The upregulation of the ecdysone signaling pathway plays an important role in microfilaria developmental transitions

Since the ecdysone signaling pathway plays a role in the morphological development of infective *D. immitis* L3 larvae, we hypothesized that the ecdysone signaling pathway may also play a role in the morphological changes observed in developing microfilaria culture. Sequence alignment of *Dim-nhr-6* and *Dim-nhr-7* with the EcRE consensus sequence identified putative EcRE in the promoter region of both genes, indicating that both could possibly act as downstream targets of the EcR (Fig. 5). Using ddPCR, we performed absolute quantification of the ecdysone signaling system gene transcripts and showed for the first time that morphologically distinct *D. immitis* L1 larvae displayed different transcript expression of the ecdysone signaling cascade. The results showed that the overall transcript expression of the ecdysone signaling system components increased gradually towards the end of the 22-day* in vitro* incubation period in both the no-cell and *An. gambiae* cell-conditioning group, with the *An. gambiae* cell-conditioning group showing higher transcript levels for *Dim-EcR*, *Dim-rxr-1*, and *Dim-nhr-6* genes (Fig. 6). Although microfilariae cultured without cells showed a higher transcript level of *Dim-nhr-7* during the beginning of the culture period, the transcript level of microfilariae cultured with *An. gambiae* cell-conditioned medium rose quickly by day 22. The finding that the overall transcript levels for the ecdysone signaling system components tended to be higher in microfilariae cultured in *An. gambiae* cell-conditioned media, which corresponded to higher numbers of developing larval stages in the* in vitro* culture, suggests that factors secreted by *An. gambiae* cell may activate the EcR and the downstream ecdysone signaling system, and that the activation of the EcR may play an important role in the morphological transition of the microfilarial stage. It would be of interest, in a future study, to attempt to identify (a) factor(s) released by the insect cells which activate the EcR.

Interestingly, while all four genes of the ecdysone signaling cascade targeted in this study displayed a similar pattern of expression over time, the large increase in the overall transcript level of *Dim-nhr-6* in developing larvae suggests that the EcR/RXR complex possibly upregulates the mRNA expression level of *Dim-nhr-6* to a much higher extent than *Dim-nhr-7*, and that *Dim-nhr-6* might be a crucial downstream target of EcR.

Given the increase in expression of genes in the ecdysone signaling pathway, it was interesting that 20E significantly increased the numbers of *D. immitis* microfilariae that developed to later stages (Fig. 7). The incubations with 20E were conducted in the absence of the insect cells or their secretions in order to establish whether 20E itself had an effect. While 20E did increase the number of larvae developed, 20E by itself did not have as dramatic an effect on the development as did the factors released by, or changed by, the mosquito cells.

Although previously published results have mentioned the sex-specific pattern of the ecdysone cascade members in *D. immitis* adults was possibly due to gravidity of the female adult worms [37–40], ddPCR results from the present study showed that expression of the ecdysone cascade members could still be detected in adult male parasites, although the mRNA levels in males were lower than those of the females (Fig. 6). This result could mean that expression of the ecdysone signaling pathway in adult females is not entirely involved in microfilaria production and that further experiments are needed to determine the role of the ecdysone signaling pathway in the female and male adult stages.

## Conclusions

To our knowledge, this study is the first to depict,* in vitro*, the detailed morphological transitions of *D. immitis* L1 larvae that occur inside the arthropod host. The present work shows* in vitro* culture conditions that allow microfilariae to develop to pre-sausage, sausage and late sausage stage L1 larvae. However, many of the later stage larvae were somewhat degenerated, development did not proceed through the L1/L2 molt, and the time it took for the development observed* in vitro* was longer than the time it takes in the mosquito. More work is needed to improve further the* in vitro* culture conditions to better mimic development in the mosquito, and perhaps the* in vitro* culture conditions will need to change as development occurs. Our results also demonstrate that factors secreted from mosquito cells play an important role in the development of *D. immitis* microfilariae and that the ecdysone cascade may contribute to the morphological changes seen with cultured microfilariae. These findings contribute to a better understanding of the developmental regulation of *D. immitis* microfilariae and open up new avenues for future investigations of the effects of ecdysone compounds and components released by mosquito cells on microfilarial development.

## Supplementary information

**Additional file 1: Table S1.** Primer sequences used to amplify *D. immitis* ecdysone signaling cascade components.

**Additional file 2: Video S1.** Movement of *D. immitis* microfilaria.

**Additional file 3: Video S2.** Movement of *D. immitis* pre-sausage.

**Additional file 4: Video S3.** Movement of *D. immitis *sausage.

**Additional file 5: Video S4.** Movement of *D. immitis* late sausage.

**Additional file 6: Video S5.** Imaging of live larva. Microfilariae were cultured with *An*. *gambiae* 4a-3B cells and imaged over time to observe if any shedding took place. Pre-sausage stage starts from 00:00:00; sausage stage starts from 00:00:16; late sausage stage starts from 00:00:21.

## Data Availability

The data supporting the conclusions of this article are included within the article.

## Abbreviations

20E: 20-Hydroxyecdysone
ddPCR: Droplet digital PCR
EcR: Ecdysone receptor
EcRE: Ecdysone-response element
HP-β-CD: (2-Hydroxypropyl)-β-cyclodextrin
L1: First-stage (larvae)
L3: Third-stage (larvae)
ML: Macrocyclic lactone
*nhr*: Nuclear hormone receptor genes
NR: Nuclear receptor
RXR: Mammalian retinoid X receptor protein
USP: Ultraspiracle protein
